# Synthesis, Characterization and Electrochemical Performance of a Redox-Responsive Polybenzopyrrole@Nickel Oxide Nanocomposite for Robust and Efficient Faraday Energy Storage

**DOI:** 10.3390/nano12030513

**Published:** 2022-02-01

**Authors:** Bushra Begum, Salma Bilal, Anwar ul Haq Ali Shah, Philipp Röse

**Affiliations:** 1 National Centre of Excellence in Physical Chemistry 1, University of Peshawar, Peshawar 25120, Pakistan; bushrachemist248@gmail.com; 2 Karlsruhe Institute of Technology (KIT), Institute for Applied Materials–Electrochemical Technologies (IAM-ET), 76131 Karlsruhe, Germany; 3 Institute of Chemical Science, University of Peshawar, Peshawar 25120, Pakistan; anwarulhaqalishah@uop.edu.pk

**Keywords:** conductive polymer, oxidative polymerization, energy storage, polymeric nanocomposite, hybrid electrode material, supercapattery

## Abstract

A polybenzopyrrole@nickel oxide (Pbp@NiO) nanocomposite was synthesized by an oxidative chemical one-pot method and tested as an active material for hybrid electrodes in an electrochemical supercapattery device. The as-prepared composite material exhibits a desirable 3D cross-linked nanostructured morphology and a synergistic effect between the polymer and metal oxide, which improved both physical properties and electrochemical performance. The unprocessed material was characterized by X-ray diffraction, FTIR and UV–Vis spectroscopy, scanning electron microscopy/energy disperse X-ray analysis, and thermogravimetry. The nanocomposite material was deposited without a binder on gold current collectors and investigated for electrochemical behavior and performance in a symmetrical two- and three-electrode cell setup. A high specific capacity of up to 105 C g^−1^ was obtained for the Pbp@NiO-based electrodes with a gravimetric energy density of 17.5 Wh kg^−1^, a power density of 1925 W kg^−1^, and excellent stability over 10,000 cycles.

## 1. Introduction

In the search for a better life, the call for electrically powered unconventional and innovative technologies is becoming louder and louder [1,2]. With the development of advanced electronic devices and rapid economic growth, the concepts of an “energy crisis” and a “sustainable energy future” are echoing worldwide [2,3]. Due to the rapidly growing demand for electrical energy, modern energy conversion and generation methods have attracted great attention from technologists and researchers [4]. Facing the increasing energy constraints of the modern world, many efforts have been made to develop emission-free, sustainable, and efficient energy conversion along with effective storage of intermittent renewable energy to replace conventional capacitors and batteries [5,6].

Supercapacitors based on faradic reactions are the most sustainable and consistent alternative for the future energy crisis. They have high power density, fast and efficient charge/discharge capability, as well as a very simple manufacturing process with a moderate energy storage capacity that is maintained for a long time compared to other energy storage devices [2,6,7,8]. Polymer-based supercapacitors also have very low weight and negligible toxicity [9]. These properties make them a suitable replacement for conventional energy storage systems. Supercapacitors cover a significant range of applications, including aerospace, hybrid vehicles, voltage stabilizers, electronics, and rail transportation [10]. Typical electrode materials for supercapacitors are composed of metal oxides such as Fe_2_O_3_, NiO, RuO_2_, Co_2_O_3_, and conjugated highly conductive polymers such as polyaniline (PANI), polypyrrole (PPy), polythiophene (PTh), etc. Conjugated polymers have come into focus due to their high capacity, low production cost, and remarkably high thermal stability [11,12]. However, the morphology of these polymers cannot be easily controlled, which has a significant impact on their electron transport properties. Polymer chain agglomeration usually imparts sluggish inter-chain tunneling as well as intra-chain hopping of electrons. This leads to reduced electrical conductivity, poor electrochemical functionality, and low mechanical stability [13]. In addition, the applicability of conjugated polymers in supercapacitors is limited by their lower solubility and poor adhesive properties, requiring a binder for electrode fabrication [12,14]. These disadvantages can be minimized by producing hybrid materials whose individual properties minimize the sum of the disadvantages and thus increase the overall performance. Therefore, numerous hybrid electrode materials and complex strategies are employed to overcome these limitations. Jinyong et al. integrated PANI with N-doped carbon nanotubes to develop a hierarchical core–shell composite based on flexible fibers that offers high performance and stability [15]. Moreover, the combination of different oxides/hydroxides with the incorporation of heteroatoms, merging metal silicates with metal oxides, hydroxides, and other materials such as carbon or reduced graphene oxide for the formation of nanostructures and their nanocomposites based on hybrid supercapacitors, has also been investigated [16,17,18,19,20,21]. However, these are complex and costly processes that result in a material with low processability and poor reproducibility, limiting their practical implementation on a large scale.

Alternatively, the combination of two pseudocapacitive materials (conjugated polymer and metal oxide) can provide a viable, highly effective, and economical way to utilize the best of both electrode materials. Hybrid materials, particularly polymer–metal oxide nanocomposite, have greatly impacted research due to their high energy density, electrical conductivity, structural diversity, and excellent cycling stability, which have great potential for future supercapacitor designs [14,22,23]. By integrating a conjugated polymer and a metal oxide into a high-quality functional and composite electrode material, the energy holding capacity and mechanical stability were significantly improved. In addition, the volume deformation of the electrode material during the charge–discharge process was also largely controlled [12,14].

Nickel oxide (NiO) is considered as the most attractive candidate for faradaic energy storage due to its high theoretical specific capacity, good redox reversibility, easy synthesis, good cyclic stability, and low cost [12,13,14,24]. Designing polymeric hybrids by integrating them with NiO could exhibit synergistic effects that provide improved morphological properties and subsequent electrochemical functionality that the single NiO or conjugated polymer cannot. Flower-like PANI/NiO and PPy/NiO nanostructured electrode materials on nickel foams have been synthesized by Bangning et al. and Wenjing et al. [25,26]. At 1 A g^−1^, PANI/NiO displayed a maximum specific capacitance (2,565 F g^−1^) with the constant capacitance retention (−100%) after 5000 cycles, whereas NiO/PPy presented a highest specific capacitance of 595 F g^−1^ at 1 A g^−1^ and 81% capacitance retention after 1000 charge–discharge cycles. Similarly, a supercapacitor electrode assembly of NiO/Ni(OH)_2_/PEDOT nano-flowers on contra wires has been reported by Yang et al. [27]. NiO/Ni(OH)_2_/PEDOT delivered a specific capacitance of 404 mF cm^−2^ at 4 mA cm^−2^ and 82% capacitance retention after 100 repetitive cycles. 

Polymeric composites with NiO not only have the advantage of improved electrochemical properties, but their electrochemically active composite electrode materials are also still limited to a few commonly cited conjugated polymers. There is still room for exploring additional redox-active polymeric composites embedded with NiO to complement the present supercapacitive materials.

Polybenzopyrrole (Pbp) is an emerging nitrogen-containing, redox-active, conjugated polymer. Pbp exhibits high thermal and electrochemical stability, but its applications as a faradaic supercapacitor electrode material are limited by its low conductivity and mechanical strength during long-term cycling [28,29]. Those features can be compensated by combination with NiO.

In this paper, a facile, cost-efficient, and convenient one-pot in situ synthesis strategy for interconnected nanostructured Pbp-integrated NiO (Pbp@NiO) hybrid nanocomposites is presented. This simple approach to Pbp@NiO resulted in better thermal stability and a simple electrode fabrication process. The synergy between Pbp and NiO provides a complementary set of morphological and supercapacitive properties for Pbp@NiO chains. The fabricated Pbp@NiO nanocomposite has been successfully used as a binder-free, redox-active electrode material for FS, providing excellent specific capacity and cycle rate capability. Moreover, our method is a scalable, economical, and template-free synthesis of Pbp@NiO nanocomposite electrodes with improved supercapacitive behavior and thus can be commercially preferred.

## 2. Materials and Methods

### 2.1. Chemicals

Pbp@NiO was synthesized from commercially available materials: benzopyrrole (C_8_H_7_N, >99%), ammonium persulfate (APS, >98%), dodecylbenzenesulfonic acid (DBSA, >95%), chloroform (>99.8%), nickel oxide (>99.9%), and acetone (99%) were purchased from Sigma-Aldrich (St. Louis, MI, USA). Sulfuric acid (98%) and toluene (99%) were obtained from Scharlau (Barcelona, CAT, Spain) and 2-Propanol (99.5%) and dimethyl sulfoxide (99.5%) from Daejung (Seoul, Korea). All chemicals were used directly without further purification. Deionized water (>18 MΩ, Merck MilliQ, Darmstadt, Germany) was used for purification and preparation of the electrolyte.

### 2.2. Proceedure for the Synthesis of the Pbp@NiO Nanocomposite

**Scheme 1:**
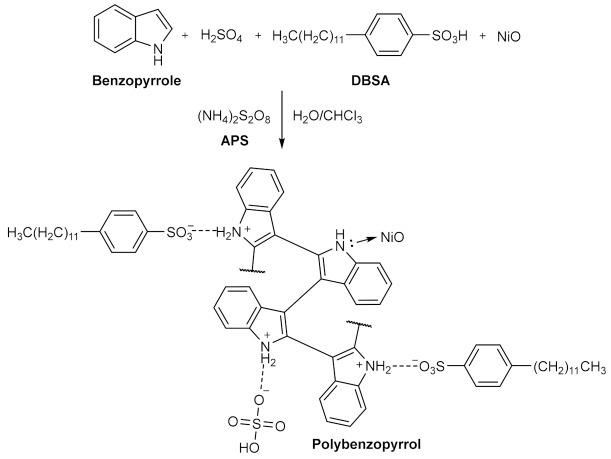
Synthesis of Pbp@NiO via anionic surfactant assisted oxidative polymerization.

The synthesis of Pbp@NiO was executed via anionic surfactant assisted oxidative polymerization (Scheme 1). In a 250 mL flask, freshly prepared H_2_SO_4_ solution (40 mL, 0.1 M) was added followed by addition of DBSA (1.0 mL), APS (7.30 mmol, 2.7 eq), and NiO (0.10 g (1.33 mmol, 0.5 eq, Pbp@NiO_0.1_), 0.20 g (2.67 mmol, 1.0 eq, Pbp@NiO_0.2_), and 0.30 g (4.0 mmol, 1.5 eq, Pbp@NiO_0.3_)) under rapid stirring (800 rpm) for 5–10 min. The polymerization was initiated by gradual addition of a solution of benzopyrrole in chloroform (0.9 M, 2.84 mmol, 1.06 eq) at room temperature and stirred for 24 h for a complete reaction. In the course of time, the mixture turned dark green. Acetone/water 1:1 (*v*/*v*) was added to the reaction mixture to precipitate Pbp@NiO. The precipitant was filtered, washed with acetone/water 1:1 (*v*/*v*) until the filtrate became colorless, and dried at 60 °C in vacuum for 24 h to give 280.0 mg (53%) Pbp@NiO_0.2_ as dark green powder.

For comparison, pure Pbp was prepared using the same procedure without adding NiO.

### 2.3. Strutural and Physicochemical Characterization

Scanning electron microscopy for structural analysis was performed on a Supra-55VP FEGSEM from ZEISS. Moreover, SEM-energy dispersive X-ray (SEM-EDX, Oxford Instrument) analysis was employed for identification of the elemental composition of the polymer sample. X-ray diffraction (XRD) analysis was carried out using a Bruker D8 Advance diffractometer with Cu Kα radiation (λ = 1.54 A) at 40 kV and 35 mA current with 2θ ranging from 10° to 80°, step width of 0.0164°, and a step rate of 1 s^−1^. Fourier transform infrared spectroscopy (FTIR) was performed on an Affinity-1S FT-IR spectrometer from Shimadzu, scanning over an effective range of 450 to 4000 cm^−1^. Optical absorption spectroscopy of Pbp@NiO in DMSO was executed with a UV–Vis spectrophotometer (Buckinghamshire, UK) in the wavelength range of 200–1000 nm. The thermal degradation patterns of Pbp@NiO nanocomposites were accomplished with a thermogravimetric analyzer (TGA, Perkin Elmer, Waltham, MA, USA). The sample was heated with a heating rate of 10 °C per minute in the nitrogen (N_2_) atmosphere.

### 2.4. Electrochemical Charaterisation in Three-Electrode Assambly 

Electrochemical experiments were carried out using a ZRA/Potentiostat/Galvanostat Reference 3000 from Gamry. For analysis of the redox-features of pure Pbp and Pbp@NiO nanocomposites, cyclic voltammetry was performed in a three-electrode setup using a polymer-coated gold sheet as working electrode and a bare gold counter electrode (each 1 × 1 cm^2^) with 1.0 M H_2_SO_4_ solution as electrolyte. For preparation of the working electrode, 0.2 mg pure Pbp or Pbp@NiO was dispersed in 2-propanol/toluene (1:2 *v*/*v*) and drop casted on a gold sheet. The coating was dried at room temperature for 10 min. The potential was referred to a Ag/AgCl (KCl_sat_ in H_2_O) reference electrode. In order to avoid hydrogen evolution reaction and obtain the safe and optimum operating potential window of the Pbp@NiO nanocomposite electrode, the potential was incremented (@ 0.1 V) from −0.4 V to various positive potentials (0.8, 0.9, 1.0, 1.1, and 1.2 V). Next, cyclic voltammograms were recorded at different scan rates of 20 to 100 mV s^−1^. The specific capacity for all nanocomposite electrodes was calculated from the CV using Equation (1) [30,31,32]:

(1) $$Q_{s}=\int_{V_{i}}^{V_{f}}I\times VdV/\left(m\times v\right)\;$$

where *Q_s_* is the specific capacity (C g^−1^), *ʋ* is the potential sweep rate (mV s^−1^), m is the mass of active electrode material (g), and $\int_{V_{i}}^{V_{f}}I\times VdV$ is area obtained from the integration of the area under the CV curve.

The electro-active surface area of the Pbp@NiO electrode was estimated by cyclic voltammetry measurements at various scan rates using 10 mM K_3_[Fe(CN)_6_] in 0.1 M KCl electrolyte solution. The effective surface area was calculated using Randles–Ševčík Equation (2) [33]:

*I_P_* = (2.687 × 10^5^) *n*^3/2^ × *ʋ*^1/2^ × *D*^1/2^ × *A* × *c* (2)

where *A* refers to the effective surface area of the electrode (cm^2^), *I_P_* is the peak current (A), *c* is the concentration of K_3_[Fe(CN)_6_], *ʋ* is the potential sweep rate, and *D* is the diffusion coefficient in 0.1 M KCl (5.7 × 10^−6^ cm^2^ s^−1^). *n* = 1 is the number of electrons transferred during the process.

Galvanostatic charge–discharge (GCD) was performed at various current densities of 0.35 to 5.0 A∙g^−1^, and the specific charge *Q_s_* (C∙g^−1^) is determined from Equation (3) [31,32,34]:

*Q_s_* = (*I* × *∆t*)/*m* (3)

where *∆t* is the discharge time (s), *m* is the mass of active electrode material (g), and *I* is the applied constant current (A). 

Electrochemical impedance spectroscopy (EIS) was used for identification of relevant processes and their contribution on the overall performance (e.g., electrical series resistance, charge-transfer processes, and mass-transport). Measurements were carried out using an excitation amplitude of 10 mV_rms_ at V_DC_ of 200 mV and a frequency range of 10^5^ to 0.05 Hz.

### 2.5. Faradaic Supercapattery Assambly and Performance Evaluation 

To understand the real electrochemical performance of Pbp@NiO_0.2_ as active material, electrochemical analysis in a symmetric two-electrode device is essential. A symmetrical supercapacitor was built by assembling two Pbp@NiO_0.2_-coated gold electrodes with a Whatman (Grade 41) separator in between connected to 5.0 mL of 1.0 M H_2_SO_4_ solution as electrolyte reservoir. CV experiments were performed at various potential sweep rates (20–100 mV s^−1^). Galvanostatic charge–discharge (GCD) experiments were performed at varying current densities between 0.5 and 5.0 A g^−1^. The *Q_s_* values were obtained from GCD curves for the symmetric two-electrode cell using Equation (3). Moreover, the gravimetric energy density, gravimetric power density, and energy efficiency of the assembled supercapattery device are given by Equations (4)–(6) [31,32,35]:

*E* = *(∆V* × *Q_s_*) / (2 × 3.6) (4)

*P* = (*E* × 3600)/*∆t* (5)

*η_E_* = (*E*_Discharge_/*E*_Charge_) × 100 (6)

where *E*, *P*, *∆V*, *Q_s_*, *η_E_*, and *∆t* are the energy density (Wh kg^−1^), power density (W∙kg^−1^), charge energy density (Wh kg^−1^), potential window (*ΔV*), specific capacity (C∙g^−1^), energy efficiency (%), and discharge time (s), respectively. A Ragone plot was created using the values of *E* and *P*. The charge–discharge cycling stability of fabricated two-electrode cell was evaluated at constant current density of 1 A∙g^−1^ for 10,000 cycles. Electrochemical impedance spectroscopy of the supercapacitor device was performed analogous to experiments in three-electrode setup.

## 3. Results and Discussion

### 3.1. Proposed Growth Mechanism of Pbp@NiO

The synthesis of Pbp@NiO is displayed in Scheme 1. The in-situ oxidation involves a radical cation polymerization of benzopyrrole facilitated by ammonium persulfate, H_2_SO_4_, DBSA, and NiO. The uniform structural growth of Pbp@NiO is supported by the DBSA. It is presumed that benzopyrrole neutral molecules undergo successive oxidation by ammonium persulfate, producing benzopyrrole radical cations. These radical cations undergo sequential addition/coupling at the C-2 and C-3 positions of benzopyrrole along with the removal of protons to produce Pbp. Subsequently, the doping process occurs simultaneously during the polymer chain formation. The negatively charged HSO_4_^−^- and DBSA^−^-anion dopant ions are attached at the protonated nitrogen atoms of the growing oxidized polymer chain to balance the charge. Simultaneously, the electrostatic charges of the Pbp cause the integration of NiO. In this way, a number of doping anions and NiO are simultaneously incorporated into the Pbp matrix.

### 3.2. Analysis of the Physico-Chemical Properties of Pbp@NiO

**Figure 1:**
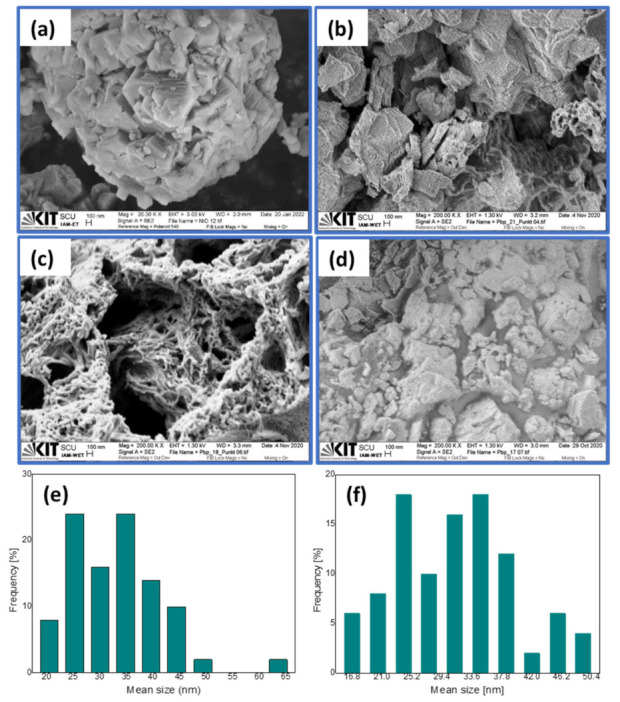
SEM images of (**a**) NiO, (**b**) Pbp@NiO_0.1_, (**c**) Pbp@NiO_0.2_, and (**d**) Pbp@NiO_0.3_, at 200 kx magnification. Size distribution of (**e**), the NiO nanoplates’ thickness, and (**f**) the diameter of the cross-section of Pbp@NiO_0.2_.

The morphology of active materials for supercapacitor applications strongly correlates with the electrochemical properties. Hollow nanostructures are desired, due to the large effective electrochemical surface and efficient fast transport processes inside the electrode. Figure 1a–d show SEM images of NiO and the synthesized Pbp@NiO nanocomposites.

NiO consists of numerous nanoplate-like structures with uneven morphological features (Figure 1a). It can be seen that the nanoplates of irregular shape are stacked together, leaving some micropores on the surface. Pbp@NiO_0.1_ consists of a collection of particles of various sizes and shapes in the mid-micro to millimeter scale, partially enclosing microporous vacancies (Figure 1b). The same is found for Pbp@NiO_0.3_, consisting of macroporous surface structure with particle sizes in the micrometer range but no vacancies (Figure 1d). In contrast, Pbp@NiO_0.2_ shows intertwined and uniformly shaped nanostructured fibers of different sizes forming a hollow structure (Figure 1c). This suggests that the amount of NiO nanoparticles exerts a direct influence on the structure of the polymer matrix, with an optimum for Pbp@NiO_0.2_. It is expected that the interconnected nanostructured morphology of Pbp@NiO_0.2_ enhances electron transport along the polymer chain. The vacancies also allow ionic interaction throughout the material and with the electrolyte. Furthermore, Nano Measurer 1.2.5 was used to determine the diameter of the cross-section of the nanofibers to obtain an estimate of the size distribution. Fifty random features from Figure 1a,c were selected and measured. Figure 1e displays the NiO nanoplates’ thickness distribution ranging between 18.6 nm and 65.1 nm with an average thickness of 32.6 nm. Similarly, a size distribution between 15.6 nm and 50.9 nm with a mean diameter size of 31.0 nm was obtained for Pbp@NiO_0.2_ (Figure 1f). The structure suggests that high specific capacity, energy retention capacity, and cycling stability can be achieved by allowing easy entry and exit of ions during the redox process [13,27,36].

In addition, the elemental composition and its distribution in the structure of the Pbp@NiO nanocomposite were investigated by EDX analysis. The EDX spectra and phase assignment are shown in the Supplementary Materials (pages S2–S4). The presence of Ni together with C, N, O, and S elements in the EDX spectra shows the successful incorporation of NiO together with DBSA and H_2_SO_4_ into the Pbp backbone of each nanocomposite. About 84% of the material consists of doped Pbp, of which about 4% is dopant, and the remaining 16% can be attributed to NiO in the Pbp@NiO_0.2_. Further, EDX mapping shows a uniform and even distribution of Ni in each sample along with N, O, C, and S throughout the polymer (see Supplementary Materials (page S5)). Thus, it can be suggested that NiO is clearly and homogeneously distributed. This is another potent evidence of the NiO integration with the Pbp. 

**Figure 2:**
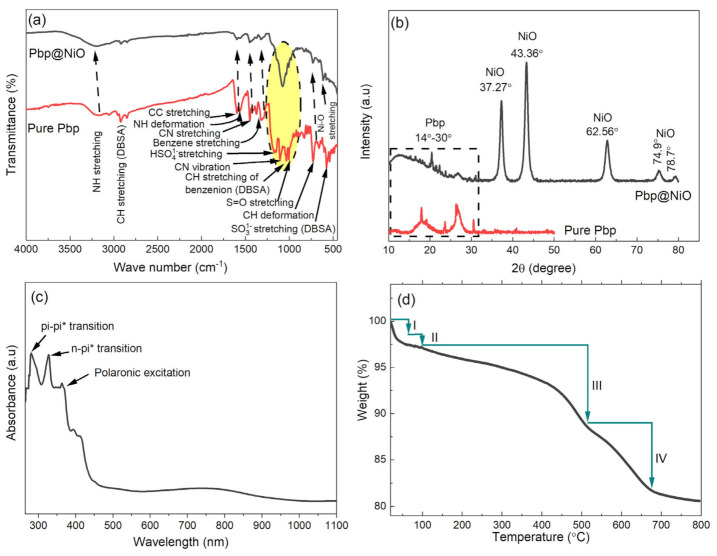
(**a**) FTIR spectra and (**b**) XRD spectra of pure Pbp and Pbp@NiO_0.2_ nanocomposite. (**c**) UV–Vis spectrum and (**d**) weight loss over wide temperature range of Pbp@NiO_0.2_ nanocomposite.

FTIR spectroscopy was carried out in order to acquire evidence regarding structural variations in the polymer after NiO incorporation in Pbp. The FTIR spectrum of pure Pbp and the Pbp@NiO_0.2_ composite is presented in Figure 2a.

All representative bands of Pbp are visible in the spectrum of Pbp@NiO_0.2_, with a shift towards longer wavenumbers compared to simple Pbp. The N-H stretching band at 3167 cm^−1^ and 1559 cm^−1^, observed in pure Pbp, is shifted to 3209 cm^−1^ and 1570 cm^−1^. From this, it can be concluded that monomeric bonding does not occur at the amine [37,38]. Compared to the pure Pbp, there is a broadening of the N-H stretching vibration with the incorporation of NiO, suggesting a coordinative donor interaction (N → Ni) between NH and NiO [39,40,41,42,43]. Besides, the bands attributed to S = O (1006 cm^−1^) and C-H stretching of the benzene ring of DBSA (1030 cm^−1^), HSO_4_^−1^ of H_2_SO_4_ (1168 cm^−1^), and C-N vibration overlapped to give shoulder bands and a strong peak stretching at 1078 cm^−1^. The observations confirm a strong association of NiO within the Pbp. Besides, the peak situated at 616 cm^−1^ corresponds to the existence of stretching of Ni-O, indicating its successful incorporation in the Pbp@NiO_0.2_ hybrid. The literature reports nickel oxides peaks in the range of 450 to 700 cm^−1^ [44].

Since SEM/EDX and FTIR analysis showed the successful synthesis of nanoscale uniformly structured Pbp@NiO, further investigations of crystallinity and structure were carried out using XRD. The XRD pattern of pure Pbp and the Pbp@NiO_0.2_ composite is shown in Figure 2b. The pure Pbp exhibited a broad band in the region 2θ = 14–27, which represents the low crystalline nature owned by the polymer. The Pbp@NiO_0.2_ composite showed a broad band in the same range 2θ = 14–27°, which is due to a low crystallinity of the polymer chain. On the other hand, the sharp peaks at 2θ = 37.3° (111), 43.4° (200), 62.6° (220), 74.9° (311), and 78.7° (222) revealed the presence of face-centered core crystalline NiO (JCPDS no. 04-0835) embedded in Pbp [44,45,46]. The appearance of characteristic diffraction peaks in the Pbp@NiO_0.2_ suggests that NiO is embedded in the Pbp host matrix and yet maintains a high degree of crystallinity. The results were similar to the reports available on conjugated polymer and NiO composites [47].

The average crystallite size of the Pbp@NiO_0.2_ composite was calculated using the Debye–Scherrer formula (*D* = *Kλ* × (*d* × cos θ)^−1^; *K* = 0.9). The determination of the mean crystallite size was carried out with representative reflections of NiO at 2θ = 37.32°, 43.4°, 62.6°, 74.9°, and 78.7° and is 11.1 nm [47].

The optical absorption spectrum of the Pbp@NiO_0.2_ nanocomposite is presented in Figure 2c. Pbp@NiO_0.2_ shows signifying absorption bands of Pbp with a slight shift in their positions and intensities. In the Pbp@NiO_0.2_ nanocomposite, the π−π* and *n*−π* transition between the valence and conduction band in benzenoid rings is observed at 277 and 324 nm. Additional absorption bands in the range between 344 and 790 nm are due to enhanced conjugation related to the interaction between the free electron pairs of nitrogen atoms inside the polymer chain and NiO and their polar excitation associated with this doped state of Pbp. Thus, the conjugated bond length of the polymer providing the path of π-electrons delocalization is increased. Similar results for the MnO_2_/Pbp and Ppb/PVA/Fe_3_O_4_ were already reported in the literature [48,49].

Thermal stability plays an essential role in the applicability of polymer composite-based electrodes in supercapacitors. At high temperatures, the loss of electrochemical charge storage is negatively affected by the exclusion of composite material from the polymer composite electrode. Therefore, the thermal stability of Pbp@NiO was investigated by TGA under a nitrogen environment, and the change of mass as a function of temperature was studied [50]. As shown in Figure 2d, the TGA curves revealed a four-step thermal degradation. The first and second steps lead to a small mass loss (−5–6%) at temperatures below 150 °C. This mass loss is attributed to the expulsion of absorbed solvent, water, and oxidant from the Pbp@NiO nanocomposite. The third mass loss (−6.5%) is associated with the removal of H_2_SO_4_, DBSA, and low molecular weight polymers/oligomers in the temperature range between 340 °C and 550 °C. In the fourth stage, carbon chain degradation starts above 550 °C, which is higher than most other materials. However, the mass loss in the last stage is still very low (8–10%), with a high residual mass retention of 81% at 800 °C. Pbp@NiO_0.2_ is thermally very resistant at high temperatures. This strongly suggests the formation of a uniform and ordered structure, with a distinct interaction of NiO with the Pbp polymer chain as well as a very distinct intramolecular interaction between the individual polymer chains [40,44].

### 3.3. Electrochemical Characterization and Performance Evaluation in Three-Electrode Setup

To evaluate the usability of the Pbp@NiO_x_ nanocomposite materials as an active material in a faradaic energy storage device, electrochemical studies, such as CV, GCD, and EIS, were performed in a three-electrode setup. 

**Figure 3:**
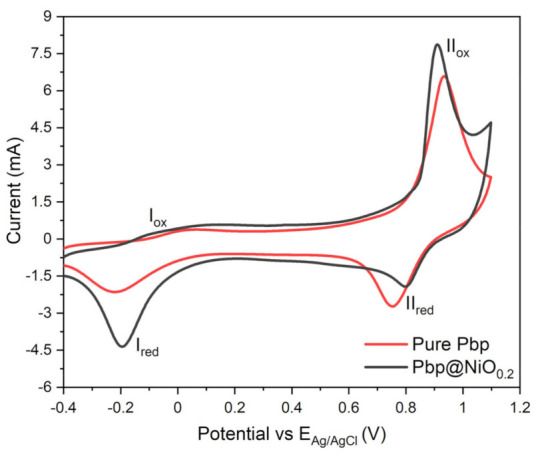
Comparative cyclic voltammograms of pure Pbp and Pbp@NiO_0.2_-coated gold electrodes in 1.0 M H_2_SO_4_ solution.

Initially, CV was performed to study the electrochemical behavior of the prepared polymers coated on the gold sheet electrode. Figure 3 displays cyclic voltammetry scans for the pure Pbp and Pbp@NiO_0.2_ nanocomposite in the potential range of −0.4 to 1.2 V. Clearly, the pure Pbp and Pbp@NiO_0.2_ nanocomposite present two pairs of sharp, well-resolved, and asymmetric/quasi-reversible redox peaks, labelled as *I*_ox_/*I*_red_ and *II*_ox_/*II*_red_. The *I*_ox_/*I*_red_, is associated with the single electron transfer of the Pbp chain, whereas the *II*_ox_/*II*_red_ is ascribed to the proton coupled electron transfer process that is initiated by protonation and deprotonation during the insertion and de-insertion of H^+^ and SO_4_^−2^ ions of electrolyte into the Pbp chain, possibly replacing the small-sized HSO_4_^−1^ (of H_2_SO_4_) and H_3_C(CH_2_)_11_(C_6_H_6_)SO_3_^−1^ (of DBSA) anions. As compared to the bulky H_3_C(CH_2_)_11_(C_6_H_6_)SO_3_^−1^ ions, the small HSO_4_^−1^ anions in the Pbp chain are expected to be replaced with SO_4_^−2^ ions of electrolyte more readily. Moreover, the existence of such redox pairs in CV due to the ion intercalation at the interface between the electrode and electrolyte is the evidence of tremendous electro-activity, excellent reversibility, and faradaic nature of both pure Pbp and Pbp@NiO_0.2_ nanocomposite electrode materials [45].

Though the CV plot of the Pbp@NiO_0.2_ nanocomposite electrode is similar to Pbp, differences in peak potentials and currents occur. The large area under the CV curve of the Pbp@NiO_0.2_ nanocomposite relative to that of pure Pbp also suggests a higher charge storage capability. For comparison, we calculated the specific capacity of the pure Pbp and Pbp@NiO_0.2_ nanocomposite electrodes at 100 mV s^−1^ scan rates using Equation (1). The specific capacity of the pure Pbp was 433 C g^−1^, while that of the Pbp@NiO_0.2_ nanocomposite resulted in 618 C g^−1^. Thus, it can be suggested that the high specific capacity, more defined peaks symmetry, and change in position and intensities of redox couples are attributed to the incorporation of NiO in the Pbp host matrix in the composite electrode.

**Figure 4:**
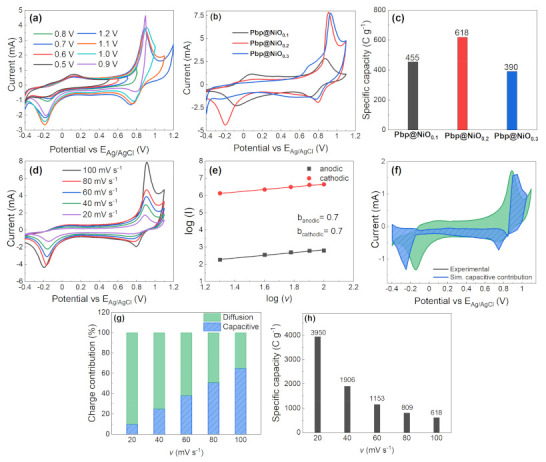
(**a**) CV spectra of Pbp@NiO_0.2_ in various potential ranges, (**b**) CV profiles of all prepared nanocomposite electrodes at 100 mV s^−1^, (**c**) comparative specific capacity of prepared nanocomposite electrodes at 100 mV s^−1^, (**d**) CV of Pbp@NiO_0.2_ as a function of scan rate, (**e**) log (*I*) as a function log (*v*), (**f**) comparative CV scans (experimental and calculated from Dunn’s equation) of Pbp@NiO_0.2_, (**g**) histogram showing the contribution of surface and diffusion capacity at various scan rates, and (**h**) specific capacity of Pbp@NiO_0.2_ electrode as a function of scan rate.

For further investigation of the redox properties, gold electrodes were coated with Pbp@NiO_x_, and cyclic voltammetry was performed initially in various potential ranges by incrementing the anodic extreme potential (from −0.4 to 0.8–1.2 V) in the steps of 0.1 V vs. Ag/AgCl (KCl_sat_ in H_2_O). The voltammograms are displayed in Figure 4a. With increasing potential window, two asymmetric redox transitions in the range of −0.20 V to 0.05 V and 0.80 V to 0.91 V can be observed. Up to 1.1 V the electrochemical behavior is reversible. As the anodic potential limit increased to 1.2 V, the redox peak currents decreased and an irreversible process was observed, which was assigned to the oxygen evolution reaction [8,49,51]. Similarly, a hydrogen evolution reaction was found at potentials at −0.4 V and below. The very low overpotential for the hydrogen evolution reaction signifies the HER is greatly suppressed by the active material and that the gold electrode support is ineffective in this cell setup. Furthermore, the occurrence of characteristic redox peaks in the Pbp@NiO nanocomposite electrodes indicates that the stable cationic state of Pbp results from the conversion of charge carriers to a delocalized polar state. These results infer that the Pbp@NiO coated electrodes are stable towards high potentials in aqueous acidic electrolyte. Therefore, the optimum operating voltage window of −0.4 to 1.1 V was selected for further electrochemical measurements. The result is consistent with the electrochemical behavior of PANI in aqueous H_2_SO_4_ electrolyte reported by Salma et al. [52]. Moreover, the separation of the two redox transitions and the shape of the U/I curve suggest that the nanocomposite behaves less like a typical supercapacitor and more like a supercapattery electrode material [30,31].

For an initial comparison of the performance, the three Pbp@NiO nanocomposites were characterized in the optimized potential window of −0.4 V to 1.1 V by cyclic voltammetry (Figure 4b). From the voltammograms, the specific capacity was calculated using Equation (1). They are 455, 618, and 390 C g^−1^ for Pbp@NiO_0.1_, Pbp@NiO_0.2_, and Pbp@NiO_0.3_, respectively (Figure 4c). The highest capacity was obtained for Pbp@NiO_0.2_. This is not very surprising, since it is generally known that pronounced 3D macrostructures with vacancies, as is the case with Pbp@NiO_0.2_, are key for high performance. Further investigations have therefore been carried out exclusively at Pbp@NiO_0.2_. 

The Pbp@NiO_0.2_ electrode was further investigated, varying the scan rates between 20–100 mV s^−1^ (Figure 4d). The area enclosed in the CV and peak currents increases linearly with the increase of scan rates from 20 to 100 mV s^−1^. The reason for this is an increase in charge mobility per time interval, which leads to a decrease in adsorption of electrolyte ions on the active material [53,54]. Moreover, the linear increase in peak current versus scan rate confirms a reversible behavior [31], whereas a slight shift of the peak potentials in the scan direction reveals a minor transport limitation of the ions to compensate for the electron neutrality during the redox transition. This is a typical behavior observed in metal oxide electrodes based on conjugated polymers [55].

For a better understanding of the present energy storage mechanism, the influence of the scan rate on peak current was further analyzed using Equation (7):

*log* (*I*) = *log* (*a*) + *b*
*log* (*v*) (7)

where the dimensionless value b predicts the expected charge storage mechanism (Figure 4e). For battery materials, b is between 0 and 0.5, whereas for supercapacitors it is between 0.8 and 1.0. Supercapattery electrodes show mean b-values of 0.5–0.8, representing a hybrid charge storage mechanism [56,57]. The Pbp@NiO_0.2_ composite electrode achieved a b-value of 0.7, implying that it is a supercapattery electrode that stores charge by both surface adsorption and diffusion mechanisms.

Furthermore, Dunn’s method was used to quantify the contribution of the capacitive (surface adsorption) and intercalation (diffusion controlled) processes to the total charge storage. The behavior of the current (I) at a fixed potential can be expressed as a combination of the two charge storage mechanisms by Equations (8) and (9) [57]:

*I*(*V*) = *k*_1_*v* + *k*_2_*v*^1/2^ (8)

*I*(*V*)/*v*^1/2^ = *k*_1_*v* + *k*_2_ (9)

where *k*_1_ and *k*_2_ are current contributions from the surface adsorption and diffusion-controlled processes, respectively. Figure 4f illustrates comparative CV scans of the experimental data and simulated data obtained by Dunn’s method. The contributions of capacitive and diffusion charge storage processes in the scan rate range of 20 to 100 mV s^−1^ are revealed in Figure 4g. At a scan rate of 20 mV s^−1^, the share of the capacitive to diffusive charge storage is 10:90%. With the increasing scan rate, the contribution increases steadily to 65:35%. Thus, it is concluded that the charge storage by the Pbp@NiO_0.2_ nanocomposite electrode is a combination of diffusion and surface capacitance.

The specific capacity of the Pbp@NiO_0.2_ electrode calculated from CVs at different potential sweep rates is shown in Figure 4h. The Pbp@NiO_0.2_ nanocomposite electrode shows a decrease in specific capacity from 3950 to 618 C g^−1^ with the increase of the scan rate from 20–100 mV s^−1^. The decrease in specific capacitance at higher potential sweep rates can be attributed to a lower penetration of the inner surface of the active material, due to increasing diffusion limitation. This leads to a reduction of the effectively used surface and thus also to a lower charge storage [6,58,59]. Nevertheless, an excellent redox activity at 100 mV s^−1^ can be retained. The redox activity and charge storage capacity of Pbp@NiO_0.2_ can be attributed to two factors. Firstly, the incorporation of NiO into the polymer structure, which significantly increased the redox properties, and secondly, the structural properties of the nanocomposite material, with its fibrous and porous composition.

**Figure 5:**
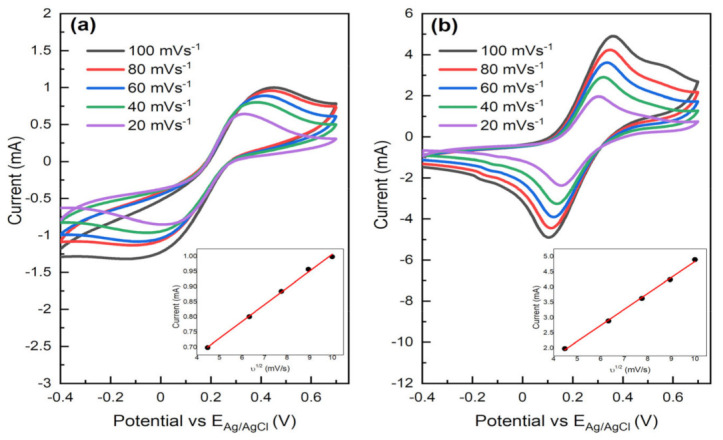
Cyclic voltammetry profiles of (**a**) gold and (**b**) Pbp@NiO_0.2_ modified gold electrodes in 10 mM K_3_[Fe(CN)_6_] and 0.1 M KCl_aq_ solution at different scan rates. Inset: corresponding anodic peak current as a function square root of scan rate.

To confirm this statement, we estimated the electrochemical effective surface area of a bare gold sheet and Pbp@NiO_0.2_ coated the gold electrode. CV scans of bare gold and Pbp@NiO modified gold electrodes are presented in Figure 5a,b. Therefore, measurements of 10 mM K_3_[Fe(CN)_6_] in 0.1 M KCl_aq_ were performed between −0.4 V and 1.0 V vs. Ag/AgCl (KCl_sat_ in H_2_O) at various scan rates. The electrochemically active surface area was determined using the Randles–Sevcik Equation (2), whereas I_p_ and ν^1/2^ were determined by the linear slope of the respective values from the CVs. For the bare gold sheet and the electrodes coated with Pbp@NiO_0.2_, the values of the slope are 0.06 and 0.52, respectively. This results in an effective surface area of 0.52 cm^2^ for the gold electrode and 2.57 cm^2^ (12,850 cm^2^ g^−1^ with regard to the used amount for coating) for the Pbp@NiO coated electrode.

**Figure 6:**
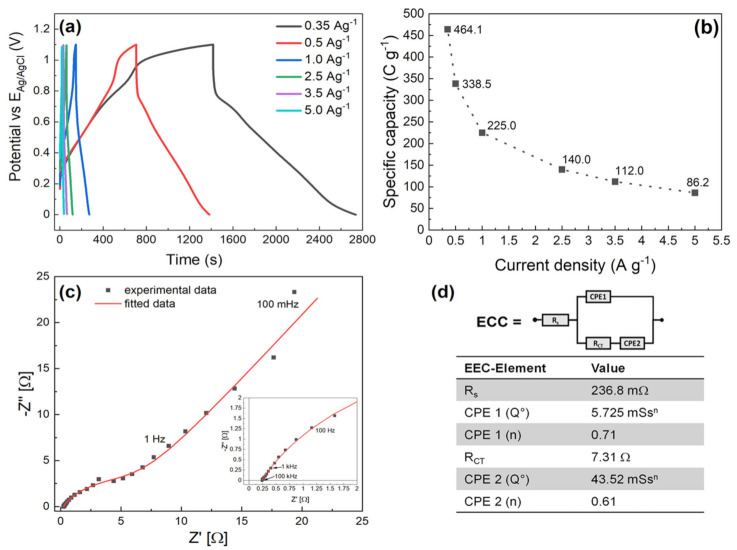
(**a**) Galvanostatic charge–discharge curves at various current densities, (**b**) respective specific capacity at various current densities, (**c**) EIS of Pbp@NiO_0.2_ nanocomposite electrode at E_DC_ = 200 mV in a frequency range of 10^5^ to 0.05 Hz. (**d**) Fitting parameters are given in the table.

For investigations of the electrode behavior under constant current loading, the Pbp@NiO_0.2_ electrode was charged and discharged at different gravimetric current densities ranging from 0.35 to 5.0 A g^−1^ in a potential range of 0 to 1.1 V (Figure 6a). The galvanostatic charge–discharge (GCD) curves have an asymmetric distorted triangular shape with an obvious plateau due to the faradaic nature of the electrode material [32]. The Pbp@NiO_0.2_ electrode exhibited a long charge–discharge time and a maximum specific capacity of 464 C g^−1^ at 0.35 A g^−1^. The high specific capacity can be explained by the porous interconnected structural properties of the polymer composite and the synergy between Pbp and NiO. Application of higher current densities was expected to result in a decrease in specific capacity, e.g., from 464 to 86 C g^−1^ with an increase from 0.35 to 5.0 A g^−1^ (Figure 6b). For current densities between 0.35 and 1 A g^−1^, a good capacity retention of 74 to 64% was obtained. At higher current densities, the specific capacity drops steadily with a linear gradient. The limitation is caused by the increase of the internal resistance during the charging and discharging processes, which is caused by the limited occupancy of the active sites in the porous electrode. Above a current density of 3.5 A g^−1^, the specific capacity value further drops to 86.2 C g^−1^. It is to be expected that above this current density, the inner surface of the polymer nanocomposite is only involved in the energy storage process to a small extent [6,51].

To gain further in-depth insights into the performance-determining electrochemical processes and their limitations, the Pbp@NiO_0.2_ nanocomposite electrode in a three-electrode cell setup was investigated by electrochemical impedance spectroscopy (Figure 6c). The measurements were performed in a frequency range between 50 mHz–10.5 kHz, at a DC potential of 200 mV, and an excitation amplitude of 10 mV_rms_.

The results of the impedance measurement are shown in the Nyquist plot in Figure 6c. The impedance spectrum can be divided into three typical ranges for supercapacitors. The first is the intersection with the horizontal axis at higher frequencies (>100 kHz), which is referred to as the series resistance (R_s_), consisting of solution resistance, current leads, and current collectors. The second region is a distorted semicircle in the mid-frequency range (100 kHz to −30 Hz), which contributes to the charge transfer resistance (R_CT_) of the Pbp@NiO_0.2_ nanocomposite. At low frequencies (<−30 Hz), an inclined straight line forming an angle (−61°) is observed, which is attributed to slow ion diffusion processes resulting in an increase in capacitive behavior. 

For a better analysis of the measured data, a simplified electrical equivalent circuit was used (Figure 6d). This is composed of the series resistance (R_s_), a resistance to describe the ohmic resistance of the charge transport (R_CT_) through the electrode and two constant phase elements CPE1 and CPE2. CPE1 was used to describe the pseudocapacitive part of the charge transfer for the inhomogeneous respective Pbp@NiO_0.2_ at various current densities and particle distributions of the nanocomposite electrode, and CPE2 expresses the ion species diffusion of the electrolyte components within the porous electrode structure. For the series resistance R_s_, a small value of 236.8 mΩ was obtained. Similarly, the active material has a low charge transfer resistance R_CT_, which is due to the fast reversibility of the redox processes at the active material/electrolyte interface. This is in alignment with the observations from the cyclic voltammetry and charge–discharge experiments. The low resistance values can be attributed to the doping of the polybenzopyrrole with NiO [51,60]. In connection with the low charge transfer resistance, there is a good value of Q*^n^* = 5.725 mSs*^n^* for the CPE1 element with a value for *n* = 0.71, which is typical for pseudocapacitive redox behavior in conductive doped polymers. This confirms that it is mainly a redox-based energy storage mechanism. CPE2 describes an almost ideal Warburg diffusion with a contribution of the exponent *n* of 0.61, which confirms the diffusion characteristics, as predicted from the CV study.

Moreover, the conductivity (*σ*, S cm^−1^) of Pbp@NiO_0.2_ was calculated from the Nyquist plot via Equation (10):

*σ* = *d* (*R* × *A*)^−1^ (10)

where *d* (cm) is the thickness of the electrode, *R* (Ω) is the bulk resistance obtained from the Nyquist plot, and *A* (cm^2^) is the cross-sectional area of the electrode. The conductivity of the Pbp@NiO_0.2_ nanocomposite electrode was 5.8 mS cm^−1^.

The data from the electrochemical impedance measurements explain the good electrochemical performance of Pbp@NiO_0.2_ and indicate its potential suitability for supercapattery applications [51].

### 3.4. Performance Analysis of Pbp@NiO_0.2_ as Active Material in a Symmetric Supercapattery Device

To explore the actual potential of the Pbp@NiO_0.2_ electrode material, a symmetrical supercapattery device with Pbp@NiO as the active material was built and investigated for its electrochemical properties and performance using CV, GCD, and EIS.

**Figure 7:**
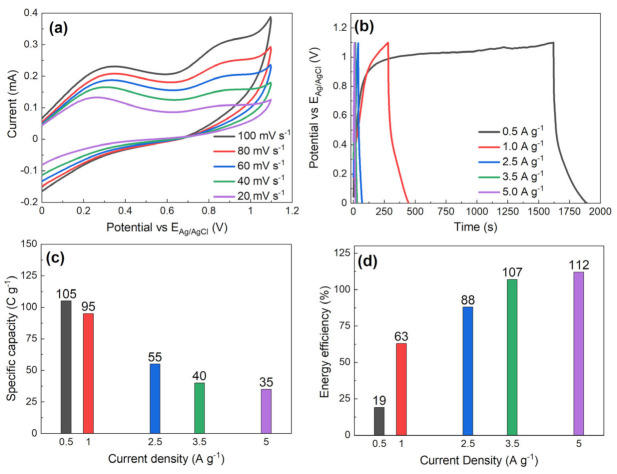
(**a**) Cyclic voltammograms at various scan rates, (**b**) GCD experiments at different current densities, (**c**) specific capacities for the GCD measurements, and (**d**) respective energy efficiencies of the symmetric Pbp@NiO_0.2_ nanocomposite supercapattery device in two-electrode setup.

In analogy to the previous experiments, CV measurements in the symmetrical two-electrode setup were investigated in a potential range from 0 to 1.1 V (Figure 7a). The scan rates were changed between 20 and 100 mV s^−1^. The results are shown in Figure 7a. The measurement curves have a non-rectangular shape with two peaks for oxidation and one reduction peak. It can be assumed that the second reduction peak (expected at −0.7–0.8 V) cannot be resolved due to the increased double layer capacitance caused by the polarization of both electrodes. An increase in the double layer capacitance is particularly evident at fast scan rates. 

As expected from the curve shapes of the CV measurements, a non-triangular shape was also found for the galvanostatic charge and final charge curves (Figure 7b). The deviation from the usual triangular curve with an almost constant potential plateau is a typical feature for supercapattery electrode material, especially in the two-electrode setup. The specific capacity was calculated according to Equation (3) (Figure 7c). It is notable that the specific capacity shows a decrease from 105 to 35 C g^−1^ with the increase in current density from 0.5 to 5.0 A g^−1^. This is due to the fact that the electrolyte ions can leave or penetrate the porous electrode structure at lower current densities. On the other hand, at high current densities, the ions interact mostly with the electrode surface; therefore, the charge-discharge time is lower, resulting in a lower specific capacity at higher current densities. This is particularly evident from the constant discharge capacity values for the current densities of 3.5 and 5 A g^−1^. Using Equation (6), the energy efficiencies at the respective current densities are obtained from the specific capacity values for the charging and discharging processes (Figure 7d). The coulombic efficiency remained about 19% at 0.5 A g^−1^ while higher current densities resulted in a steady increase up to a steady value of 112% above 3.5 A g^−1^. The higher energy efficiency at high current densities is attributed to the distortable insertion/de-insertion at the interface between the Pbp@NiO_0.2_ electrode and electrolyte at high current densities [45,61].

**Figure 8:**
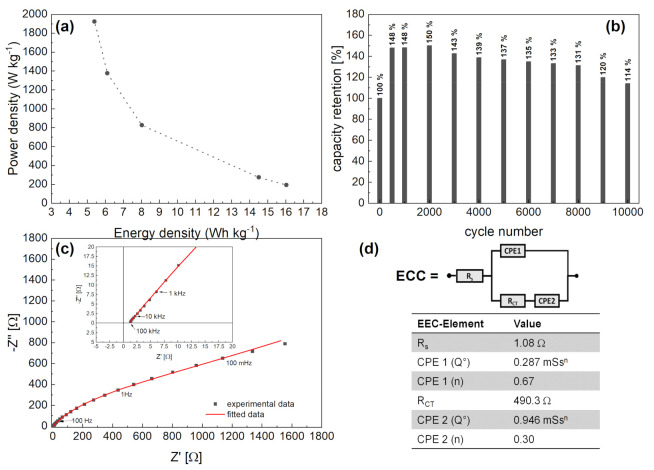
(**a**) Ragone plot (top left-0.5 A g^−1^, bottom right-5.0 A g^−1^), (**b**) cycling stability, and (**c**) EIS of the symmetric Pbp@NiO_0.2_ supercapattery assembly at E_DC_ = 200 mV in a frequency range of 10^5^ to 0.05 Hz. (**d**) Electrical equivalent circuit (EEC) and fitting parameters are given in the table.

In addition to the typical performance parameters, such as capacity values and capacity retention, key indicators such as gravimetric energy and power density help in the evaluation of active materials for electrodes for specific areas of application. For the Pbp@NiO_0.2_ symmetric supercapattery device, an inverse relationship between power and energy density can be observed, whereas the applicability at low and high current densities is limited by the gravimetric energy density (Figure 8a). A maximum energy density of 17.5 Wh kg^−1^ is achieved at a power density of 192.5 W kg^−1^ at 0.5 A g^−1^ and maintained 5.4 Wh kg^−1^ at 1925.0 W kg^−1^ at 5.0 A g^−1^. The optimal application range for the Pbp@NiO_0.2_ supercapacitor is between a specific current density of 1.0 A g^−1^ and 2.5 A g^−1^.

The results demonstrate that Pbp@NiO_0.2_ is a potential candidate for high power density applications with the necessity of a substantial energy density [49,62,63]. For better comparison, a comparison of similar supercapacitors and supercapattery devices has been made in Table 1.

In addition to energy and power density, electrochemical cycle stability is a decisive factor for the selection of suitable materials for energy storage applications. Therefore, the Pbp@NiO_0.2_ supercapattery device was processed at a current density of 1 A g^−1^ over 10,000 charge and discharge cycles. As shown in Figure 8b, capacity retention initially increased by 50% during the first 2000 cycles. The increase was explained by a surface change due to mechanical as well as electrical stress, which led to an increase in the electrochemically active surface. Over the remaining 6000 cycles, the capacitance then slowly decreased by about 22% due to natural material degradation, resulting in a loss of active surface area [64]. After 8000 cycles, increased degradation set in, resulting in a capacity retention of 114% with respect to the initial capacity after 10,000 cycles.

**Table 1:** Estimated values of electrochemical parameters of various reported conjugated polymer composites as positive electrodes.

Electrode Material	Max. E (Wh kg^−1^)	Max. *p* (W kg^−1^)	Stability/Cycles	Ref.
Pbp/Nd_2_O_3_	8.9	1000	97%/5000 cycles (at 3 A g^−1^)	[65]
Pbp/Gd_2_O_3_	<8.9	1000	85%/5000 cycles (at 3 A g^−1^)	[65]
Pbp/MoS_2_	6.0	2497	87%/5000 cycles (at 5 A g^−1^)	[66]
Cobalt oxide/Ag/PANI	14.01	1650	121.03%/3500 cycles (at 2 A g^−1^)	[31]
Manganese dioxide-PANI	17.9	261	84.7%/5000 cycles (at 2 A g^−1^)	[67]
NiCo_2_S_4_/PANI	54.06	27.1	84.5%/5000 cycles (at 8 A g^−1^)	[68]
Pbp@NiO	17.5	1925	114%/10,000 (at 1 A g^−1^)	This work

As with the three-electrode setup, the symmetrical Pbp@NiO_0.2_ device was investigated for its relevant and performance-dominant processes using electrochemical impedance spectroscopy. The device was analyzed using a perturbation amplitude of 10 mV_rms_ at a DC potential of 200 mV in a frequency range of 100 kHz to 50 mHz. The results are presented in a Nyquist plot (Figure 8c).

Compared to the three-electrode system, the electrochemical properties are less resolved in the symmetrical two-electrode design. The intersection with the horizontal axis at higher frequencies (>100 kHz) is related to the series resistance (R_s_), consisting of solution resistance, current leads, and current collectors. The implied flattened semicircle in the mid-frequency range (100 kHz to −30 Hz) is attributed to the charge transfer resistance (R_CT_) at the Pbp@NiO_0.2_/electrolyte interface. At low frequencies (<−30 Hz), a monotonically increasing straight line with an angle of −30° can be observed. An electrical equivalent circuit was used to determine relevant parameters for the description of the electrochemical processes (Figure 8d). The series resistance R_s_ was 1.08 Ω, whereas for the charge-transfer resistance R_CT_ a high value of 490 Ω was determined. The very small electrolyte and high charge transfer resistances show the fast reversibility and reduced ESR at the electrode/electrolyte interface as well as a high charge-transfer turnover of the polymer nanocomposite itself. For CPE1, a value of 0.287 mSs*^n^* with an expression of the exponent *n* = 0.67 was found, implying a strong pseudocapacitive behavior. For the transport processes of the electrochemically active species through the constant-phase-element CPE2, a value of 0.946 μSs*^n^* with a value for the exponent *n* of 0.32 was determined. This corresponds to a Warburg diffusion, with an inductive influence due to surface processes of the oppositely polarized NiO-dopant in the Pbp backbone and can be understood as a measure for an efficient and little-hindered diffusion of the active species through the charge transfer channels of the Pbp@NiO_0.2_ nanocomposite material [57]. 

## 4. Conclusions

In this work, an efficient one-step synthesis for NiO-doped polybenzopyrroles via in situ chemical oxidation was presented. The synthesis conditions led to the formation of interconnected nanostructured Pbp@NiO_0.2_ fibers with distinct vacancies. FTIR and XRD analyses showed that there was a strong interaction between the Pbp backbone and NiO dopant. This interaction was identified as a key feature determining the physical and electrochemical properties. The Pbp@NiO_0.2_ nanocomposite material had an exceptionally high thermal stability, which makes it interesting for possible high-temperature applications. Furthermore, the nanocomposite showed excellent performances for faradaic energy storage in three- and two-electrode compartments, with a specific capacity up to 105 C g^−1^ at 0.5 A g^−1^ in the symmetric two-electrode cell setup. Analysis of the Ragone plot at different current densities revealed that a symmetrical supercapattery device consisting of a Pbp@NiO_0.2_ active material is best suited for applications requiring high power densities (up to 275 W kg^−1^) at medium energy densities (−17.5 Wh kg^−1^). The experimental results obtained in this study show that the composite of polymer and metal oxide can provide a robust and high-performance composite material. The obtained Pbp@NiO_0.2_ nanocomposite can be used in supercapattery devices directly or as an active material or additive in other energy storage devices.

## Data Availability

The data presented in this study are available upon request from the corresponding author.

## Supplementary Materials

The following supporting information can be downloaded at: https://www.mdpi.com/article/10.3390/nano12030513/s1. EDX analysis of Pbp@NiO0.1 (S2), Pbp@NiO0.2 (S3) and Pbp@NiO0.3 (S4); Elemental mapping of Pbp@NiO0.1, Pbp@NiO0.2 and Pbp@NiO0.3 (S5).
