# Supplementary material for: Synthesis, Characterization and Electrochemical Performance of a Redox-Responsive Polybenzopyrrole@Nickel Oxide Nanocomposite for Robust and Efficient Faraday Energy Storage
Source: Nanomaterials (Basel). 2022 Feb 1;12(3):513. doi: 10.3390/nano12030513 (PMC8840143; doi:10.3390/nano12030513)

Supplemanetary Materials

Synthesis, Characterization, and Electrochemical Performance of a Redox-Responsive Polybenzopyrrole@Nickel Oxide Nanocomposite for Robust and Efficient Faraday Energy Storage

Bushra Begum [1], Salma Bilal [1,2,*], Anwar ul Haq Ali Shah [3] and Philipp Röse [2,*]

[1] National Centre of Excellence in Physical Chemistry 1, University of Peshawar, Peshawar 25120, Pakistan; bushrachemist248@gmail.com
[2] Karlsruhe Institute of Technology (KIT), Institute for Applied Materials–Electrochemical Technologies (IAM-ET), 76131 Karlsruhe, Germany
[3] Institute of Chemical Science, University of Peshawar, Peshawar 25120, Pakistan; anwarulhaqalishah@uop.edu.pk
* Correspondence: salmabilal@uop.edu.pk (S.B.); philipp.roese@kit.edu (P.R.)

Content	Pages
EDX analysis of Pbp@$NiO_{0.1}$	S2
EDX analysis of Pbp@$NiO_{0.2}$	S3
EDX analysis of Pbp@$NiO_{0.3}$	S4
Elemental mapping of Pbp@$NiO_{0.1}$, Pbp@$NiO_{0.2}$ and Pbp@$NiO_{0.3}$	S5

EDX analysis of Pbp@NiO$_{0.1}$, Pbp@NiO$_{0.2}$ and Pbp@NiO$_{0.3}$

Pbp@NiO$_{0.1}$:

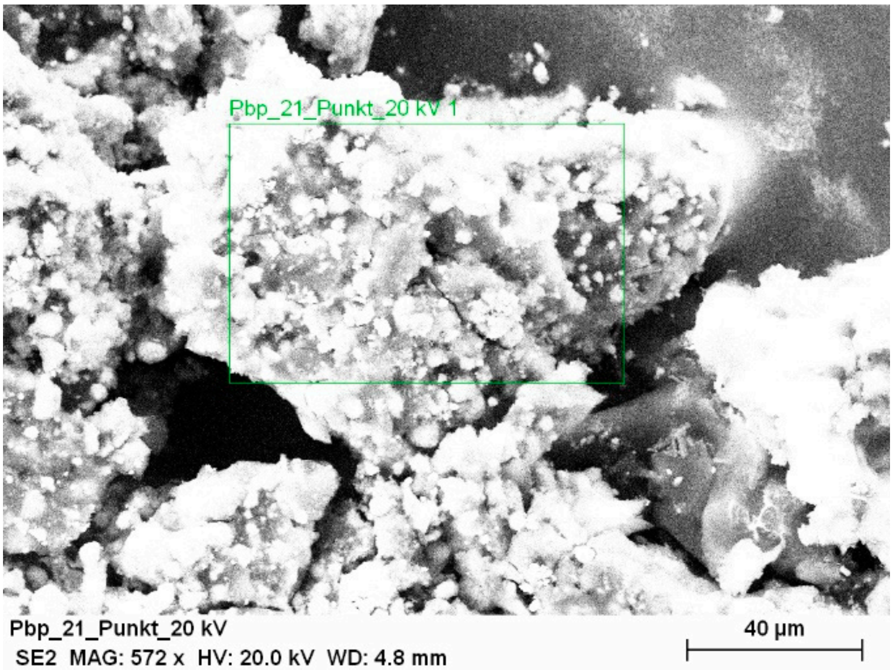

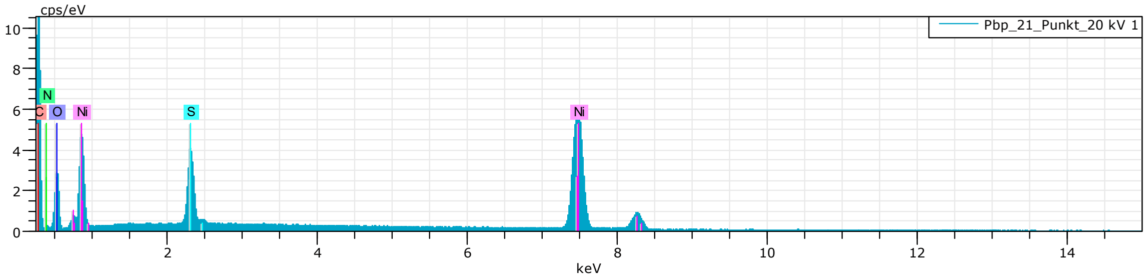

Element	AN	Series	Unn C [wt%]	Norm. C [wt%]	Atom C [wt%]	Error [%]
C	6	K-series	21.98	25.86	47.61	2.6
N	7	K-series	3.82	4.49	7.09	0.8
O	8	K-series	14.44	16.99	23.48	1.9
S	16	K-series	5.38	6.33	4.37	0.2
Ni	28	K-series	39.38	46.32	17.45	1.1

Pbp@$NiO_{0.2}$:

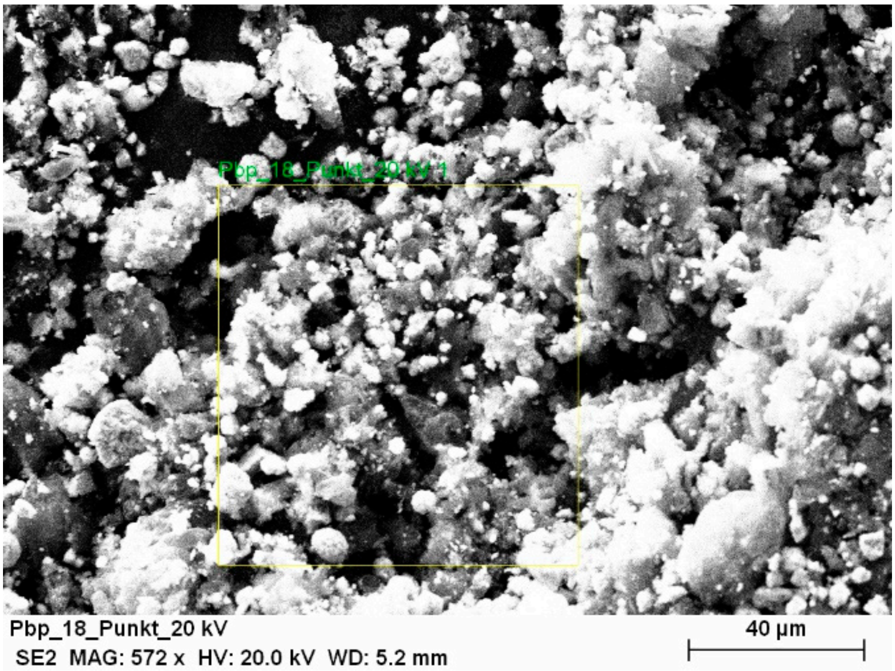

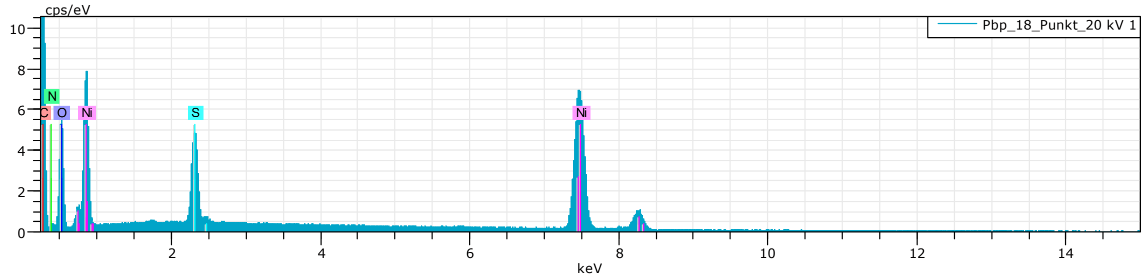

Element	AN	Series	Unn C [wt%]	Norm. C [wt%]	Atom C [wt%]	Error [%]
C	6	K-series	19.89	22.44	40.68	2.3
N	7	K-series	4.52	5.10	7.94	0.9
O	8	K-series	20.49	23.12	31.47	2.6
S	16	K-series	4.65	5.24	3.56	0.2
Ni	28	K-series	39.09	44.10	16.36	1.1

Pbp@NiO$_{0.3}$:

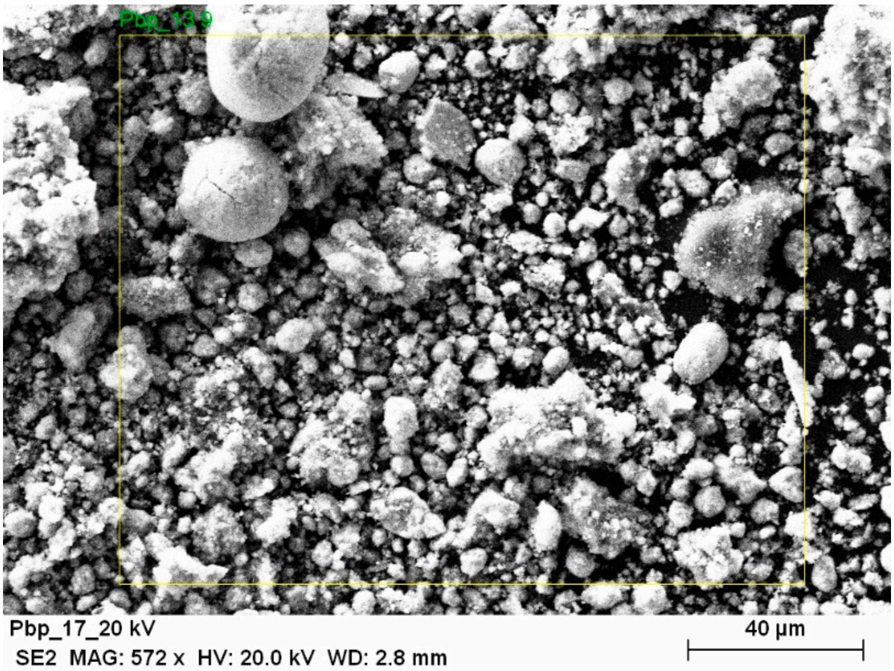

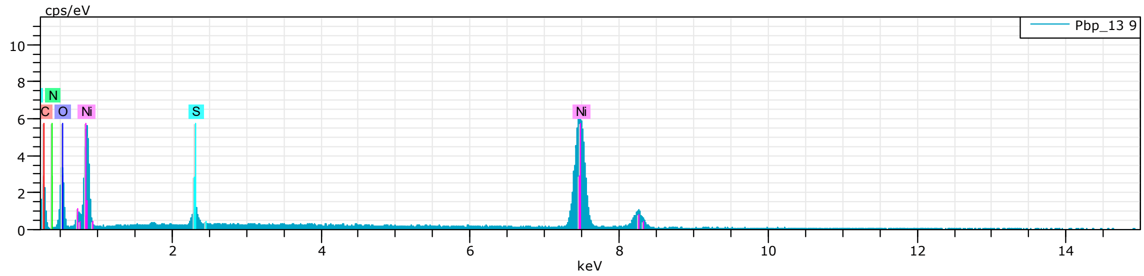

Element	AN	Series	Unn C [wt%]	Norm. C [wt%]	Atom C [wt%]	Error [%]
C	6	K-series	7.90	9.28	25.06	1.3
N	7	K-series	0.41	0.48	1.10	0.3
O	8	K-series	13.12	15.41	31.24	2.1
S	16	K-series	2.28	2.68	2.71	0.1
Ni	28	K-series	61.43	72.16	39.88	1.7

Elemental mapping of Pbp@$NiO_{0.1}$, Pbp@$NiO_{0.2}$ and Pbp@$NiO_{0.3}$

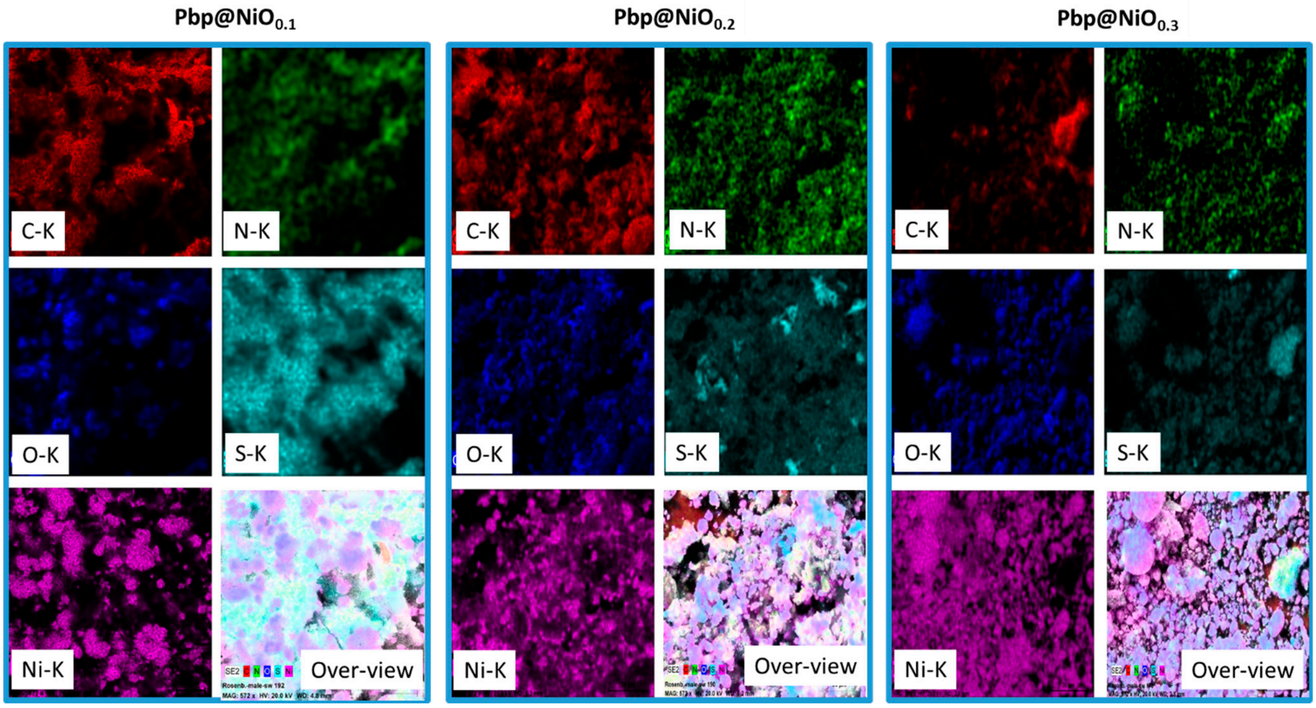

Supplement: Supplementary file 1 [file nanomaterials-12-00513-s001.zip › nanomaterials-1528665-supplementary.pdf]
